# Complete Genome Sequences of Gram-Negative Opportunistic Pathogens Isolated in Hospitals in Almaty, Kazakhstan

**DOI:** 10.1128/MRA.00974-21

**Published:** 2021-11-18

**Authors:** Ilya S. Korotetskiy, Ardak B. Jumagaziyeva, Bahkytzhan Kerimzhanova, Oleg N. Reva, Sergey V. Shilov, Tatyana Kuznetsova, Natalya Zubenko, Nadezhda Korotetskaya, Aimana Bekmukhamedova, Ganiya Satylgankyzy, Nailya G. Klivleyeva

**Affiliations:** a Scientific Center for Anti-Infectious Drugs, Almaty, Kazakhstan; b Centre for Bioinformatics and Computational Biology, Department of Biochemistry, Genetics, and Microbiology, University of Pretoria, Pretoria, South Africa; c Department of Vascular Surgery of the Syzganov National Scientific Center of Surgery, Almaty, Kazakhstan; d The Research and Production Center for Microbiology and Virology, Almaty, Kazakhstan; University of Maryland School of Medicine

## Abstract

The problem of nosocomial infections is growing due to the introduction of new treatment regimens involving immunosuppressive drugs. The genomes of seven Gram-negative clinical isolates of Escherichia, Klebsiella, and Pseudomonas were sequenced and analyzed in this study to serve as model microorganisms to study drug-induced antibiotic resistance reversion.

## ANNOUNCEMENT

Escherichia coli, Klebsiella pneumoniae, and Pseudomonas aeruginosa are the most common agents of nosocomial infections. The emergence of nosocomial pathogens often involves the acquisition of a virulence plasmid, horizontal gene transfer, and adaptive mutations. The dynamics of these processes require constant monitoring.

Seven strains of Gram-negative bacteria were isolated at the Department of Vascular Surgery of the Syzganov National Scientific Center of Surgery in Almaty, Kazakhstan. Isolates were obtained by direct plating from biological material onto selective and differential diagnostic media (Table 1). The aim of the study was to identify and perform genotyping of the potential agents of nosocomial infections. For more details on the isolates, see BioProject accession number PRJNA754843. This study was approved by the Committee of Institutional Animal Care and Use at the Scientific Center for Anti-Infectious Drugs (SCAID), Almaty.

**TABLE 1:** Deposited complete genome sequences of Gram-negative isolates

Strain name	Sample type	Media	Total no. of reads	*N*_50_ (bp)	Resistance^*a*^	MLST^*b*^	Serotype	Replicon(s)	Length (bp)	GC content (%)	Reference genome GenBank accession no.	GenBank accession no.	SRA accession no.
Escherichia coli strain SCAID WND1-2021 (1/128)	Swab from wound	CHROMagar Orientation, Endo agar	136,334	12,221	AMX, CTR, CZ, OX, E, AZM, AMP, СВ,^*c*^ АK^*c*^	ST43	H4:O25	Chromosome	5,449,567	50.56	CP041581	CP082831	SRX11971100
								Plasmid	186,443	52.21	LS992193	CP082832	
E. coli SCAID WND2-2021 (3/145)	Swab from wound	CHROMagar Orientation, Endo agar	170,744	11,412	AMX, OX, E, AMP, AZM^*c*^	ST3	H18:O17	Chromosome	5,134,206	50.77	CP046396	CP082827	SRX11971101
								Plasmid 1	139,267	51.5	CP044193	CP082828	
								Plasmid 2	106,249	49.56	CP003290	CP082829	
								Plasmid 3	32,040	41.4	CP021735	CP082830	
Klebsiella pneumoniae SCAID PHRX1-2021 (13/97)	Swab from pharynx	CHROMagar Orientation, Endo agar	72,020	11,598	AMX, OX, E, AZM, AMP	ST23	Kp1	Chromosome	5,498,275	57.43	CP026021	CP082805	SRX11971103
								Plasmid	217,781	50.16	MF993442	CP082806	
K. pneumoniae SCAID PHRX2-2021 (20/245)	Swab from pharynx	CHROMagar Orientation, Endo agar	132,267	10,935	AMX, OX, E, AMP	ST380	Kp1	Chromosome	5,319,600	57.54	CP076322	CP082796	SRX11971105
								Plasmid 1	162,135	50.25	MZ156797	CP082797	
								Plasmid 2	95,203	49.97	FO834904	CP082798	
Pseudomonas aeruginosa SCAID TST-2021 (7/157)	Swab from tracheostomy tube	CHROMagar Orientation, cetrimide agar	61,010	10,499	AMX, CTR,^*c*^ CZ, OX, E, AZM,^*c*^ IPM, AMP	ST308		Chromosome	7,173,620	65.81	NZ_CP027172	CP082823	SRX11971107
P. aeruginosa SCAID WND1-2021 (9/195)	Swab from wound	CHROMagar Orientation, cetrimide agar	142,460	11,343	AMX, CTR, CZ, OX, E, AMP, IPM^*c*^	ST244		Chromosome	7,093,992	65.9	CP032257	CP082822	SRX11971108
P. aeruginosa SCAID PLC1-2021 (16/222)	Pleural cavity	CHROMagar Orientation, cetrimide agar	79,229	11,145	AMX, CZ, OX, E, AZM, AMP	ST308		Chromosome	7,124,329	65.85	CP027172	CP082821	SRX11971109

a АK, amikacin; AMP, ampicillin; AMX, amoxicillin; AZM, azithromycin; СВ, carbenicillin; CTR, ceftriaxone; CZ, cefazolin; Е, erythromycin; IPM, imipenem; OX, oxacillin.

b MLST, multilocus sequence typing.

c Intermediate resistance. The resistance to antibiotics was determined experimentally. The susceptibility was evaluated by the disk diffusion method in Mueller-Hinton agar (HiMedia, India). The results of the threshold inhibition zones were evaluated according to the CLSI.

For DNA extraction, cultures were grown on nutrient agar (HiMedia) for 24 h at 37°C. DNA was extracted using the PureLink genomic DNA minikit (Invitrogen, USA). DNA was sheared using the Megaruptor 3 shearing kit. A library was prepared using the PacBio SMRTbell Express template prep kit v2.0. SMRTbell templates were annealed using the Sequel binding and internal control kit v3.0. The Sequel sequencing kit v3.0 and a single-molecule real-time (SMRT) cell 1M v3 tray were used for sequencing. For each SMRT cell (Pacific Biosciences), 600 min movies were captured by Macrogen (Seoul, South Korea) using the PacBio Sequel I sequencing platform. Peaks smaller than 8 kb were removed using the BluePippin system. The numbers of generated reads and *N*_50_ values for each sample are shown in Table 1. Further processing of the DNA reads was performed using software tools as described below, with default parameter settings if not indicated otherwise. The DNA reads were quality controlled and checked for remaining adapters using LongQC v1.2.0c (1) and assembled using Canu v2.0 (2). Plasmid contigs were identified using Platon v1.6 (3). The contigs were scaffolded and joined using MeDuSa at http://combo.dbe.unifi.it/medusa (4) by comparison with the most closely related reference genomes identified in GenBank by BLASTN (Table 1). The original DNA reads were mapped to the scaffolds using pbmm2 (SMRT Link v10.10.119588) for error correction, and consensus sequences were generated from the alignments using the gcpp Arrow algorithm (SMRT Link v10.10.119588). The consensus sequences were annotated using the RAST server (https://rast.nmpdr.org/) with the RASTtk algorithm (5) and the “Fix frameshifts” setting. The chromosomal sequences were rotated to start with *dnaA* on the positive strand, and the plasmid sequences were shifted for 50 kb to perform circularization, final error fixation, and genome completion by another round of mapping of the initial PacBio reads using pbmm2. The final consensus sequences of the complete genomes were generated from the alignments and deposited at NCBI (Table 1). The GenBank annotation robot PGAP was used for annotation of the deposited genomes. Multilocus sequence typing (MLST) was performed using the BIGSdb (https://bigsdb.pasteur.fr/) and CBS (https://www.cbs.dtu.dk/services/MLST) databases (6, 7). The E. coli serotypes were predicted by genotype using SerotypeFinder v2.0.1 (http://cge.cbs.dtu.dk/services/SerotypeFinder/) (8).

The E. coli isolates belonged to MLST ST43 and ST3, which are widely distributed uropathogens (9). K. pneumoniae ST23 and ST380 are abundant hypervirulent and multidrug-resistant variants which emerged due to the acquisition of pLVPK-type virulence plasmids (10–12). Two P. aeruginosa isolates belong to ST308, which is a common causative agent of nosocomial infections (13).

### Data availability.

The genome sequences are available from NCBI under BioProject accession number PRJNA754843 and the accession numbers shown in Table 1.
